# Comparative physiological and coexpression network analyses reveal the potential drought tolerance mechanism of peanut

**DOI:** 10.1186/s12870-022-03848-7

**Published:** 2022-09-26

**Authors:** Jingyao Ren, Pei Guo, He Zhang, Xiaolong Shi, Xin Ai, Jing Wang, Chunji Jiang, Xinhua Zhao, Xibo Liu, Haiqiu Yu

**Affiliations:** College of Agronomy, Shenyang Agricultural University, Shenyang, China

**Keywords:** Peanut, Drought stress, WGCNA, O_2_^•−^ /TBARs accumulation, Transcriptional regulation

## Abstract

**Background:**

Drought stress has negative effects on plant growth and productivity. In this study, a comprehensive analysis of physiological responses and gene expression was performed. The responses and expressions were compared between drought-tolerant (DT) and drought-sensitive (DS) peanut varieties to investigate the regulatory mechanisms and hub genes involved in the impact of drought stress on culture.

**Results:**

The drought-tolerant variety had robust antioxidative capacities with higher total antioxidant capacity and flavonoid contents, and it enhanced osmotic adjustment substance accumulation to adapt to drought conditions. KEGG analysis of differentially expressed genes demonstrated that photosynthesis was strongly affected by drought stress, especially in the drought-sensitive variety, which was consistent with the more severe suppression of photosynthesis. The hub genes in the key modules related to the drought response, including genes encoding protein kinase, E3 ubiquitin-protein ligase, potassium transporter, pentatricopeptide repeat-containing protein, and aspartic proteinase, were identified through a comprehensive combined analysis of genes and physiological traits using weighted gene co-expression network analysis. There were notably differentially expressed genes between the two varieties, suggesting the positive roles of these genes in peanut drought tolerance.

**Conclusion:**

A comprehensive analysis of physiological traits and relevant genes was conducted on peanuts with different drought tolerances. The findings revealed diverse drought-response mechanisms and identified candidate genes for further research.

**Supplementary Information:**

The online version contains supplementary material available at 10.1186/s12870-022-03848-7.

## Background

Peanut (*Arachis hypogaea* L.) is an important crop for both its oil and economic importance. It is widely cultivated in the tropics and subtropics around the world [1]. China is one of the largest producers of peanuts, along with India, Nigeria, and Sudan, and the number of acres planted is increasing annually [2, 3]. However, as a result of climate change, drought stress has been the most destructive factor for plant development, and it is predicted to become more frequent and intense [4, 5]. Current evidence suggests that compared with wet conditions, crop yields are reduced by as much as 25% under dry conditions, and a significant correlation between the drought index and yield has been observed in various crops, such as soybean, rice, wheat and maize [6].

When confronted with drought stress, crops undergo a range of morphological, physiological, and molecular processes. During the early phase, plants minimize water deficit by regulating stomatal aperture. Meanwhile, photosynthesis decreases due to stomatal limitation during this stage [7]. With increasing drought stress, the overaccumulation of reactive oxygen species (ROS) triggers oxidative response reactions. ROS are generated in chloroplasts, peroxisomes, mitochondria, the endoplasmic reticulum and the plasma membrane [8]. Excessive ROS attack biological macromolecules and cause increased membrane permeability, ion leakage, and chlorophyll destruction [9, 10]. To avoid the deleterious effects of ROS, plants have evolved a series of sophisticated enzymes to maintain intracellular redox state homeostasis, such as catalase, ascorbate peroxidase, glutathione peroxidase, and glutathione reductase [11].

Additionally, osmotically active substances, such as prolines, soluble sugars, sorbitol and lycine, accumulate in plant cells. The accumulation of osmotically active substances is known to be beneficial for the maintenance of cell turgor, and this process is considered the prime drought stress adaptive engine [12, 13]. An increasing body of evidence suggests that these substances contribute to soil moisture capture and sustain photosynthesis by adjusting osmotic potential [14, 15]. Additionally, osmotically active substances are beneficial for surface-bound water stabilization and sustaining the spatial structure of biological macromolecules [16].

In response to drought stress, plants possess complex processes to alter the expression of numerous genes with different functions [17]. Some genes, such as protein kinase and transcription factors, are reportedly involved in the drought response through signaling cascades and transcriptional regulation. There are many types of protein kinases. Plants sense and receive extracellular signals by cellular membrane-localized receptor-like kinases, and the leucine-rich-repeat receptor-like kinase (LRR-RLK) family is the largest receptor kinase in plants [18, 19]. They function in cell expansion, stomata development and stress response [20]. Moreover, genes encoding late embryogenesis abundant protein, antioxidants, and osmotin are considered the second category of drought response genes, and they provide protection to cellular membranes and other proteins under drought stress [21]. In addition, genes involved in water uptake and ion transport also inform drought resistance strategies [22].

Over the years, transcriptomics has been extensively applied to gene regulation network investigations in plant drought responses. It is well-recognized that weighted gene coexpression network analysis (WGCNA) can cluster highly correlated genes into modules to examine the associations between genes and traits [23]. WGCNA has been used to identify hub genes in crops such as rice, wheat and maize in response to abiotic stress [24–26]. Herein, we performed a comprehensive analysis of the gene expression patterns and physiological indices of peanut drought response by WGCNA and identified candidate genes associated with drought tolerance improvement.

## Result

### Drought-induced ROS accumulation and physiological responses in peanut

**Fig. 1:**
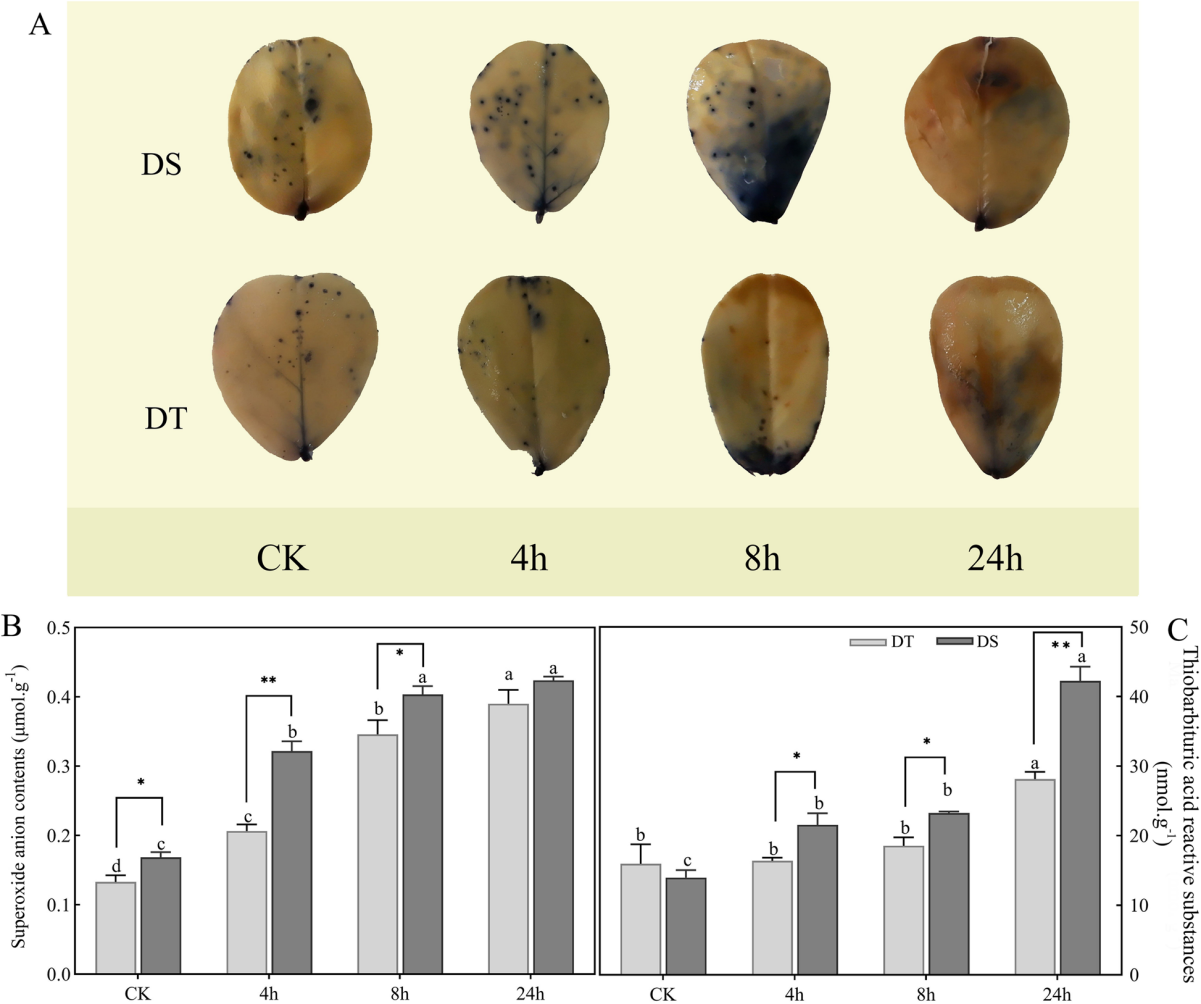
O_2_^•−^ and TBARs accumulation in peanut leaves under drought stress. **A** NBT staining in peanut leaves; **B** O_2_^•−^ accumulation in peanuts caused by drought stress. **C** TBARs contents in peanuts under drought stress. Different letters indicate significant differences at the *p* < 0.05 level between different points for each genotype, * (*p* < 0.05) and ** (*p* < 0.01) indicate significant differences between two genotypes at the same treatment point

To ascertain the oxidative stress damage induced by drought stress, the accumulation of ROS was examined in the drought-tolerant (DT) and drought-sensitive (DS) varieties. Nitro Blue Tetrazolium (NBT) staining demonstrated a significant accumulation of superoxide anion (O_2_^•−^) in peanut leaves (Fig. 1A), and O_2_^•−^ staining was more obvious in DS than in DT, increasing gradually over time in both varieties. The results were consistent with the findings of the O_2_^•−^ content determination, as shown in Fig. 1B. In DS, the thiobarbituric acid reactive substances (TBARs) content consistently increased and was significantly higher than that in DT. It increased by 76.32 and 200.74% in DT and DS after 24 h of drought stress compared with the control, respectively (Fig. 1C).

**Fig. 2:**
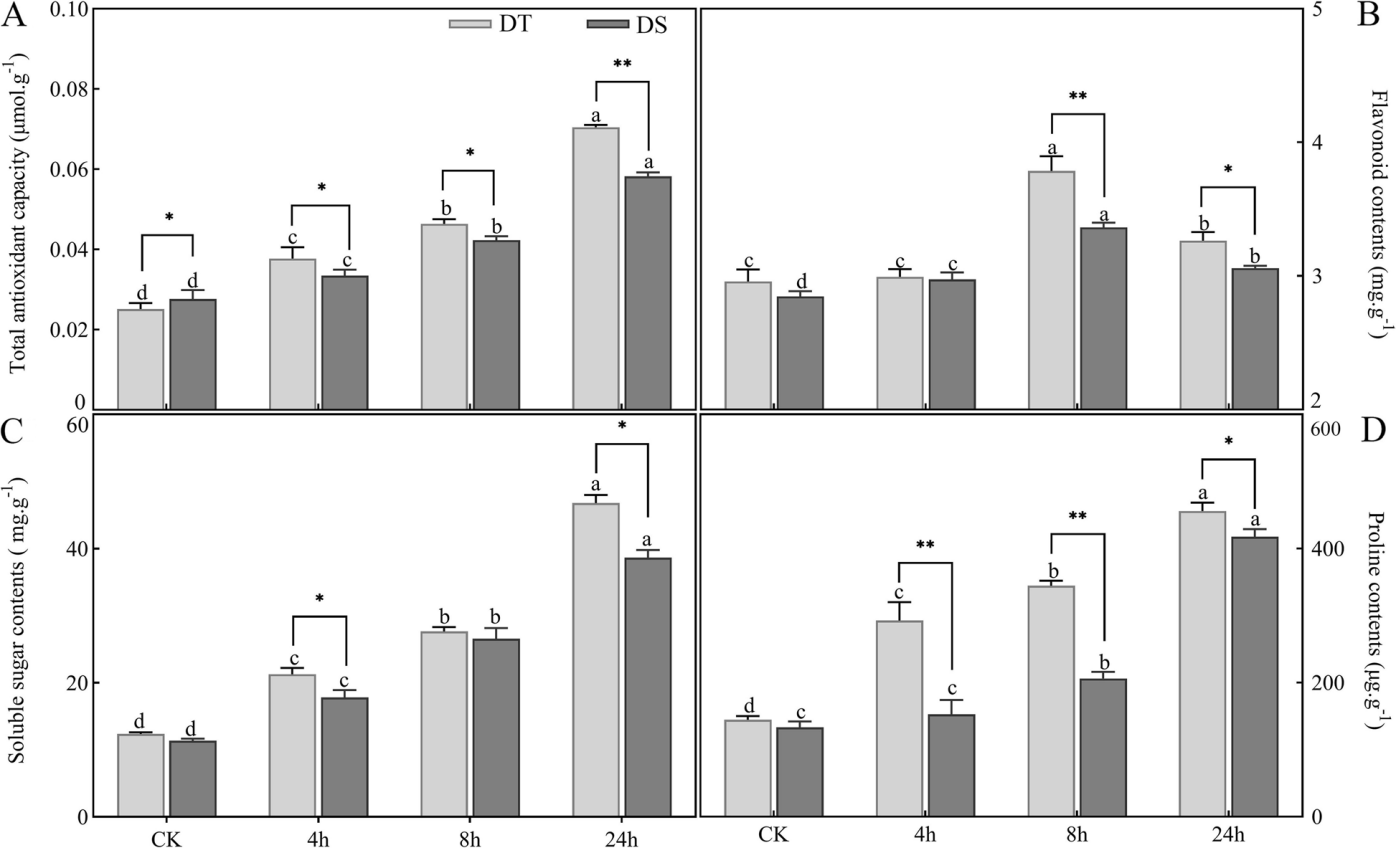
Physiological responses in DT and DS under drought stress. **A** Total antioxidant capacity; **B** Flavonoid contents; **C** Soluble sugar contents; **D** Proline contents. Different letters indicate significant differences at the *p* < 0.05 level between different points for each genotype, * (*p* < 0.05) and ** (*p* < 0.01) indicate significant differences between two genotypes at the same treatment point

Subsequently, the physiological responses were compared between the two peanut varieties. With the prolongation of stress time, increased total antioxidant capacity (T-AOC) activity, soluble sugar contents and free proline contents in leaf tissues were observed in both cultivars, and a more significant increase was observed in the drought-tolerant cultivar (Fig. 2A, C-D). In addition, quantification of flavonoid contents showed that it peaked dramatically at 8 h and then decreased at 24 h both in DS and DT, while the value of the latter variety was higher than in the former by 12.56% (Fig. 2B).

### Photosynthetic characteristics and chlorophyll fluorescence parameters

**Fig. 3:**
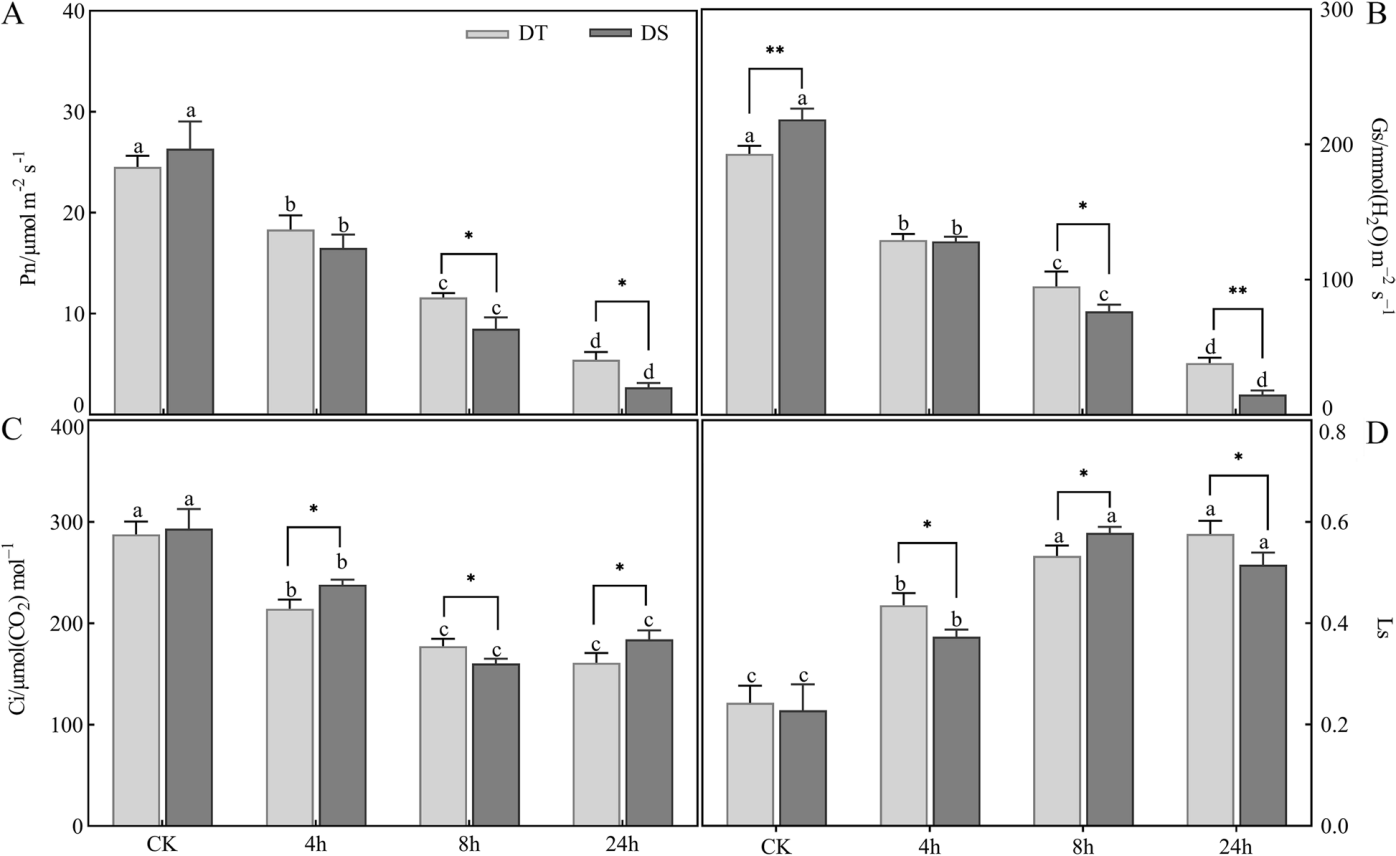
Photosynthetic characteristics in DS and DT under drought stress. The letters A-D represent Pn, Gs, Ci and Ls, respectively. Different letters indicate significant differences at the *p* < 0.05 level between different points for each genotype, * (*p* < 0.05) and ** (*p* < 0.01) indicate significant differences between two genotypes at the same treatment point

To explore the photosynthesis change of peanut when exposed to drought stress, the net photosynthetic rate (Pn), stomatal conductance (Gs), intercellular CO_2_ concentration (Ci) and stomatal limitation (Ls) were taken into consideration. As expected, at the point of 24 h drought stress, the Pn, Gs, and Ci decreased by 89.7, 93.1 and 37.3% in DS, and decreased by 77.9, 80.1 and 44.3% in DT, respectively. There were significant differences between DS and DT (Fig. 3A-C). In addition, the Ls gradually increased during stress in the two varieties, and the increase was even more obvious in DT at 4 h and 24 h (Fig. 3D). These results indicated that drought stress caused disturbances to peanut photosynthesis.

**Fig. 4:**
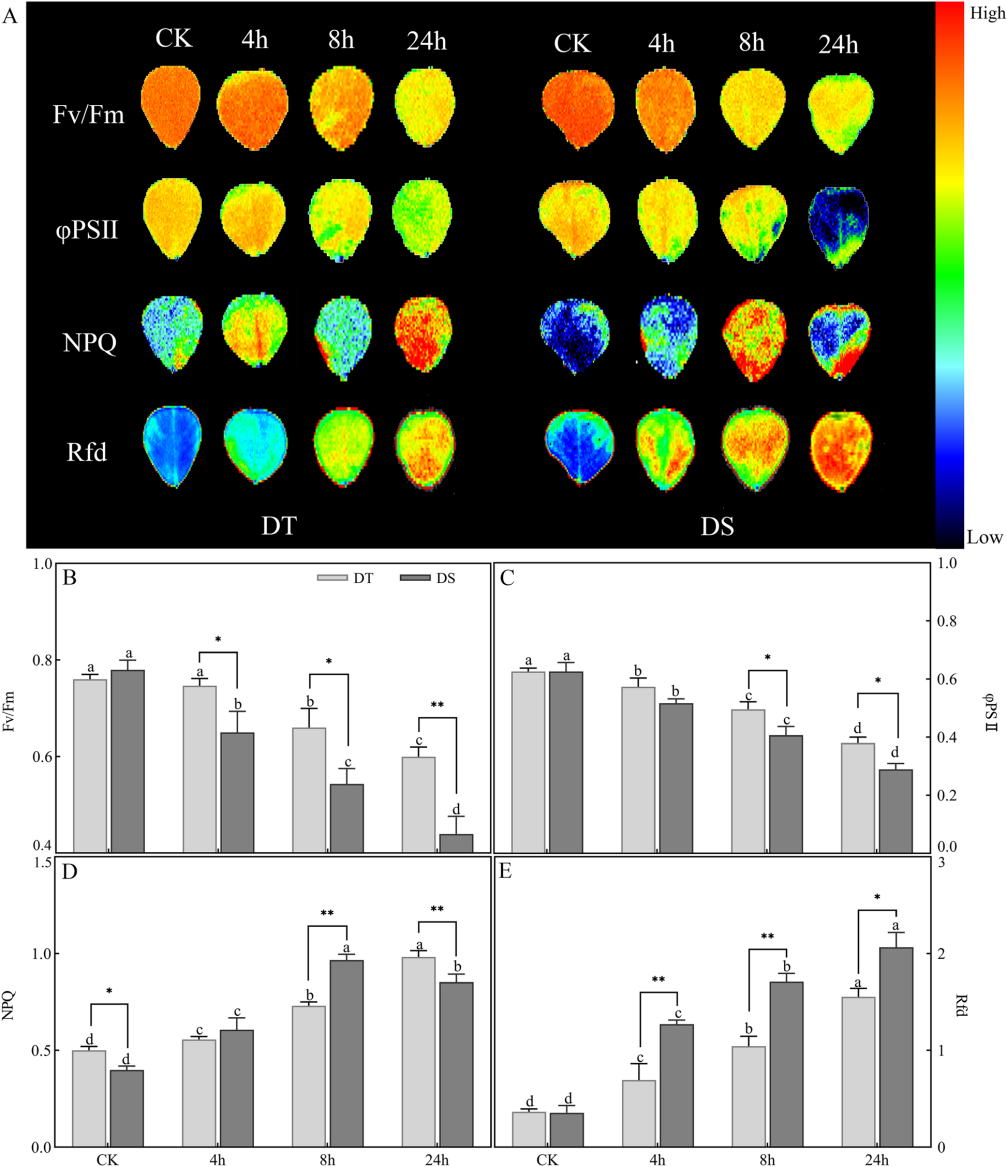
Change in chlorophyll fluorescence parameters in DS and DT under drought stress. Different letters indicate significant differences at the *p* < 0.05 level between different points for each genotype, * (*p* < 0.05) and ** (*p* < 0.01) indicate significant differences between two genotypes at the same treatment point

Furthermore, we analysed the chlorophyll fluorescence parameters in both peanut varieties subjected to drought conditions. The maximal quantum yield of PSII photochemistry (Fv/Fm) and effective quantum yield of PSII photochemistry (φPSII) decreased for both varieties under drought conditions, but the decrease was more pronounced for DS (43.58 and 55.76%) than for DT (23.40 and 38.07%). However, after drought stress, the nonphotochemical quenching coefficient (NPQ) increased by 113.33 and 96.67% in DS and DT at 24 h, respectively, compared with the control. Similarly, a significant increase could be observed in steady-state fluorescence decay rate (Rfd) in two varieties (Fig. 4).

### Comparative transcriptional profiling of two peanut varieties

To understand the transcriptomic changes induced due to drought stress, we performed RNA-Sequencing (RNA-Seq) analysis of DS and DT at 0 h, 4 h, 8 h and 24 h after the initiation of drought stress. Approximately 177.69 Gb of clean data were generated from 24 libraries, with a Q30 rate range of 94.62–95.35% (Supplementary Table S1). The clean data were aligned to the specified reference genome to obtain the mapped data, and 94.47–97.49% of reads were mapped to the reference genome. In addition, the FPKM distribution of each library was analyzed among libraries. Approximately 30.04–36.22% of genes expressed low FPKM ranging from 0 to 1. Approximately 45.86–54.67% and 14.25–20.09% of genes expressed FPKM values ranging from 1 to 10 and 10 to 100, respectively (Supplementary Fig. S1).

**Fig. 5:**
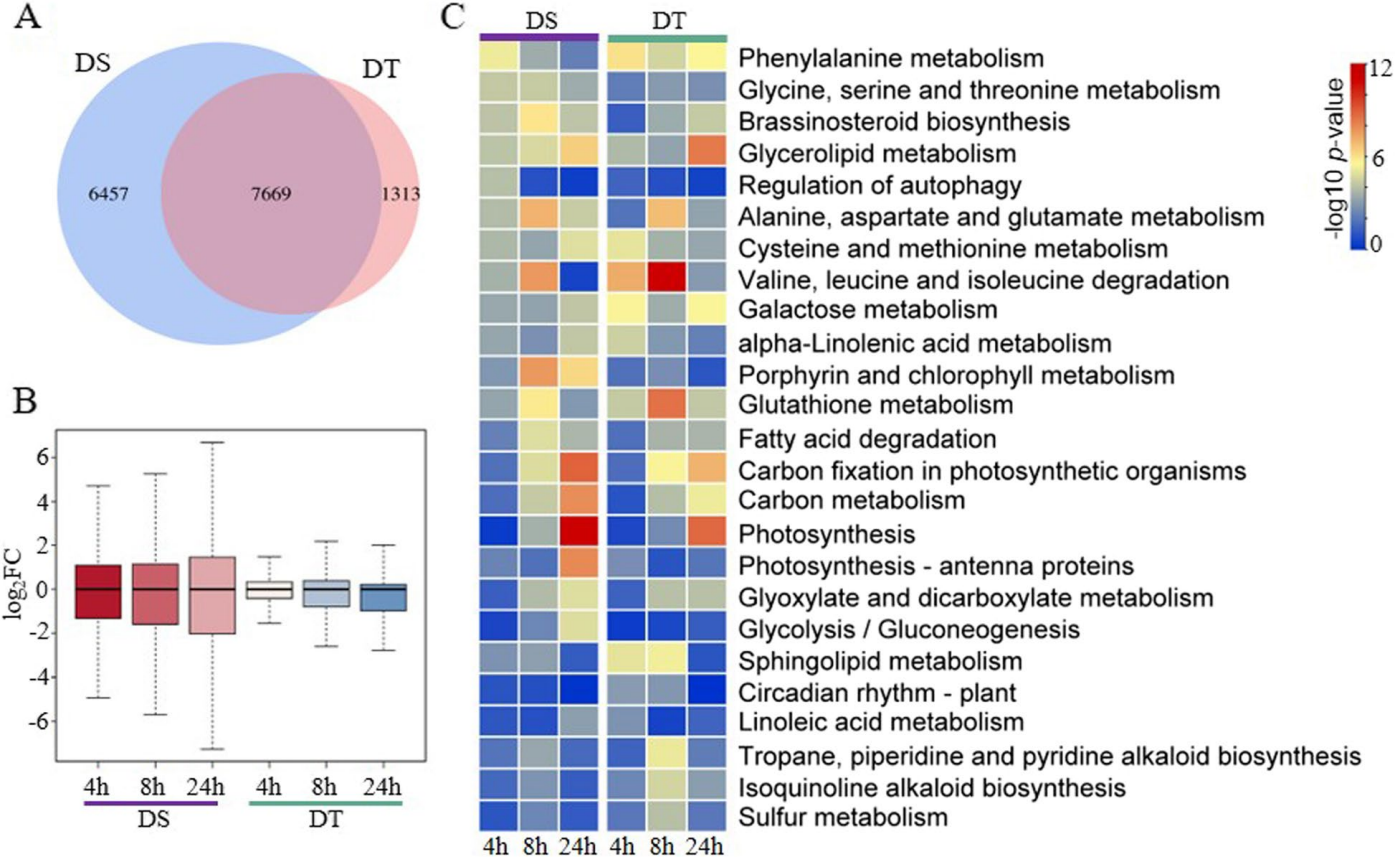
Comparative analysis of differentially expressed genes under drought stress. **A** Venn diagram of DEGs between DS and DT. **B** The expression levels of DS and DT under drought stress; **C** KEGG enrichment of DEGs in the two varieties

In total, 14,126 and 8982 genes were differentially expressed in DS and DT, respectively, with at least one sampling point based on the threshold of fold change (FC) ≥ 2 and false discovery rate (FDR) < 0.01, including 7669 common differentially expressed genes (DEGs). Moreover, the DEGs increased gradually with prolonged stress time in both varieties, and more downregulated genes were detected (Fig. 5A-B, Supplementary Table S2). To further elucidate the mechanism underlying the drought response in peanuts, Kyoto Encyclopedia of Genes and Genomes (KEGG) pathway comparisons were conducted between DS and DT [27]. As shown in Fig. 5C, carbon fixation in photosynthetic organisms, carbon metabolism, and photosynthesis pathways in both peanut cultivars were significantly enriched at the 8 h and 24 h points. Phenylalanine metabolism, galactose metabolism, and sphingolipid metabolism pathways were significantly enriched in DT, while porphyrin and chlorophyll metabolism and photosynthesis-antenna protein pathways were enriched in the DS. These results indicated that drought stress substantially affected peanut photosynthesis.

To verify the accuracy of the transcriptome, 10 mRNAs were selected randomly for qRT–PCR. Overall, the trend of genes validated by qRT–PCR was consistent with the transcriptome sequencing results, confirming the high credibility and accuracy of the transcriptome sequencing data (Supplementary Fig. S2).

### Construction of the coexpression network

**Fig. 6:**
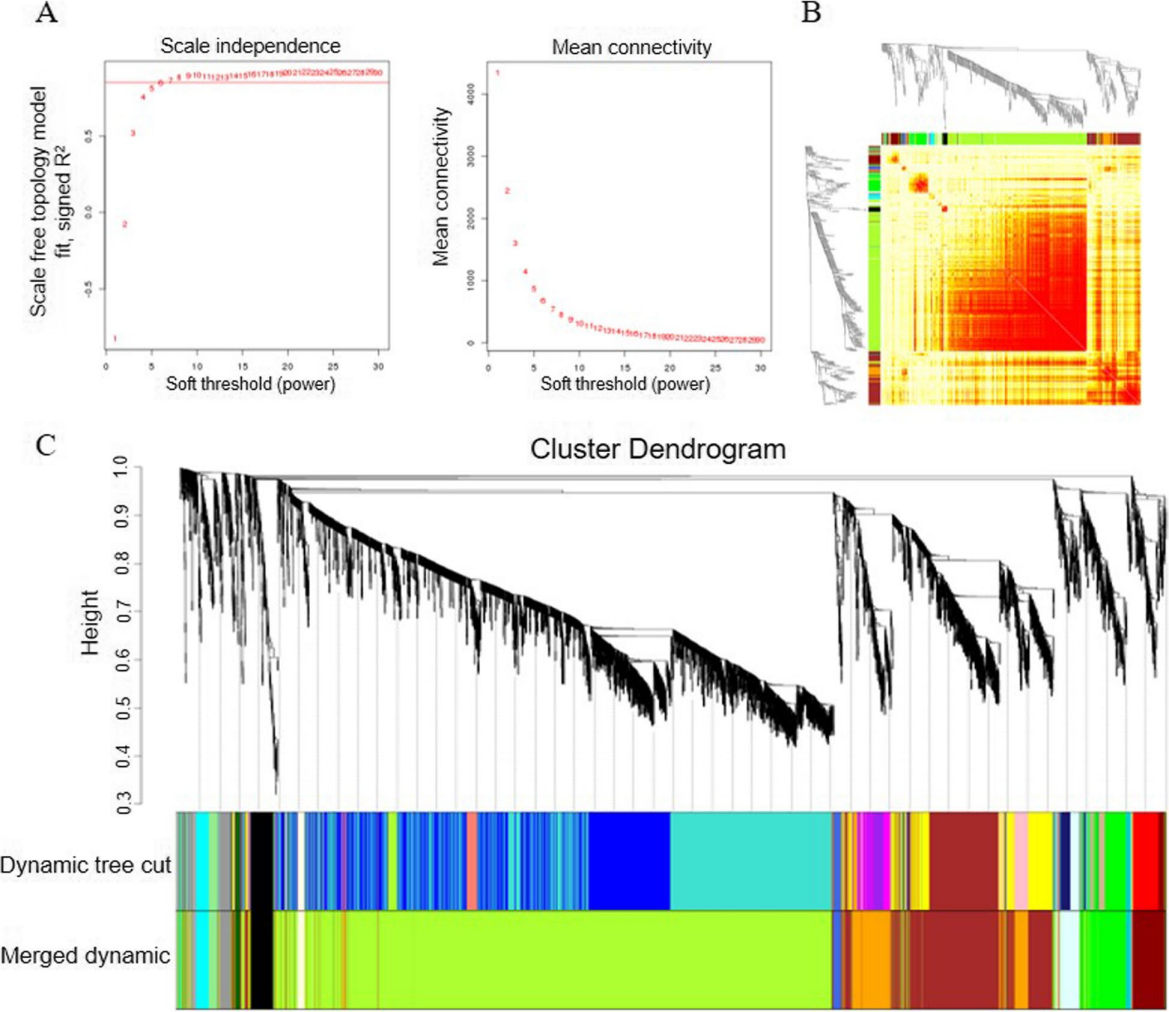
WGCNA of effectively expressed genes. **A** Scale-free topology model and mean connectivity; **B** Correlation heatmap of gene coexpression networks; **C** Hierarchical cluster tree showing coexpression modules identified by WGCNA

To obtain a comprehensive understanding of the gene regulation network under drought stress, weighted gene coexpression networks were constructed. In this work, 56,974 effectively expressed genes with FPKM≥1 in at least one library were retained for WGCNA (Supplementary Table S3). Coexpression networks were constructed at the soft-thresholding parameter of power β = 7, and genes in the same cluster were highly interconnected and had high correlation coefficients (Fig. 6A-B). As a result, 16 major tree branches were identified as individual modules through the dynamic tree cut method labelled with different colours (Fig. 6C).

### Analysis of module-trait correlation and identification of key coexpression modules

**Fig. 7:**
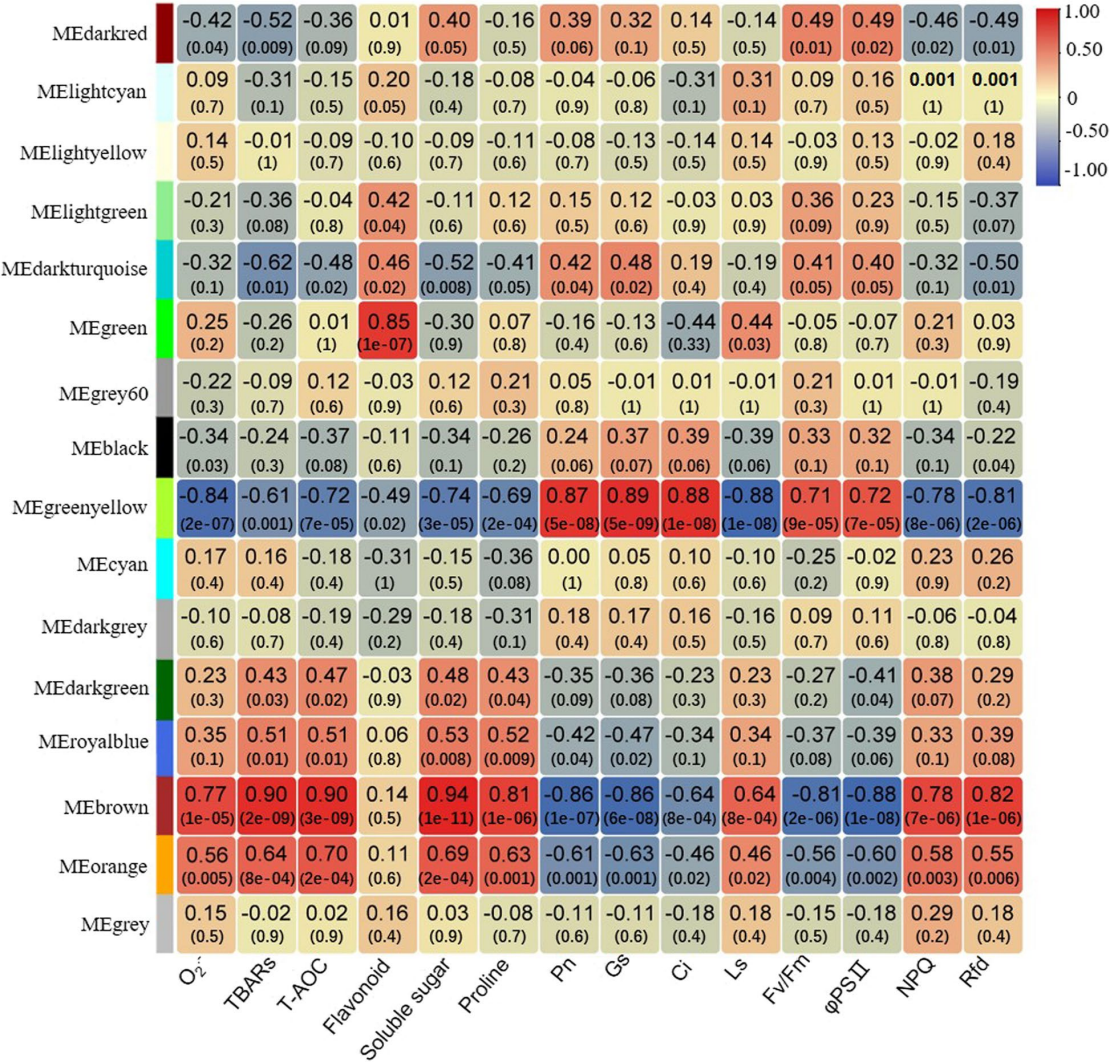
Module-traitstrait correlations and corresponding *P* values

To investigate the associations between modules and biological traits, correlations between modules and the abovementioned physiological traits were analysed. As shown in Fig. 7, there was a significant relationship between several modules and traits, such as module brown and module orange, which were significantly positively correlated with TBARs contents, T-AOC, soluble sugar and proline content and negatively correlated with Pn, Gs and Fv/Fm. The green module was strongly positively correlated with flavonoid contents. In addition, five modules, including darkred, darkturquoise, greenyellow, darkgreen, and royalblue, were significantly correlated with several specific physiological traits. Therefore, the eight modules abovementioned were further analysed.

Then, the expression profiles of the above eight modules were analysed to identify the key coexpression modules related to drought stress. Genes in the same module had similar expression patterns (Supplementary Fig. S3), and we compared the patterns between the two varieties in the same module. Genes in the orange, greenyellow and brown modules were expressed at similar levels between DS and DT after drought stress. Conversely, in the darkred, darkturquoise, and green modules, the expression patterns varied considerably among the two varieties, and higher expression levels could be observed in DT (Supplementary Fig. S3). It is speculated that the genes in these three modules are beneficial for enhancing peanut drought tolerance. As a result, the three modules, including darkred, darkturquoise, and green, were considered key coexpression modules.

### GO enrichment analysis and hub gene identification of key modules

**Fig. 8:**
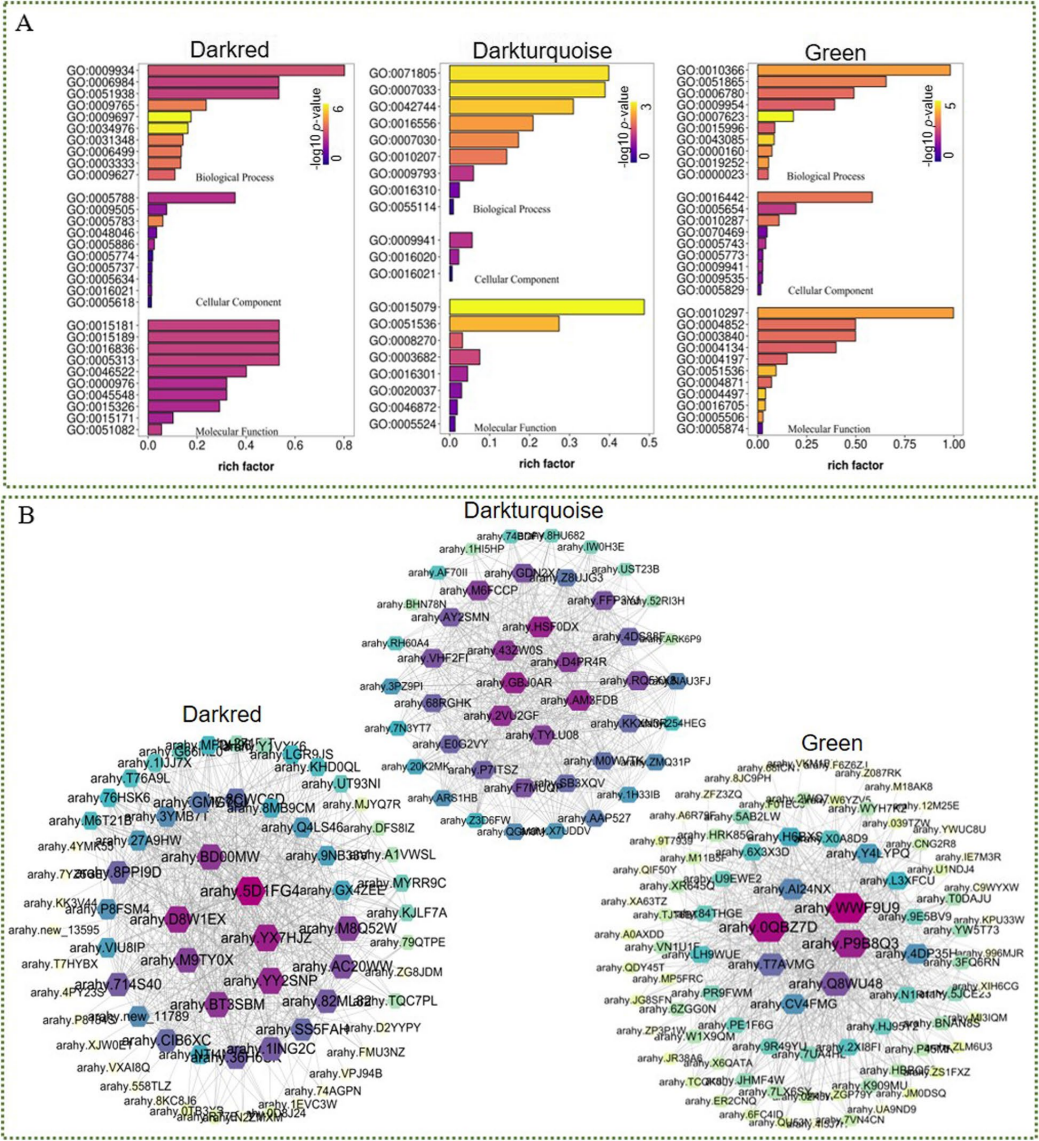
Analysis of key modules identified in peanut under drought stress. **A** GO enrichment of darkred, darkturquoise, and green modules. **B** The coexpression network and hub gene identification. The size of the nodes represents the connection degrees in the network

Gene Ontology (GO) enrichment analysis on the module genes was performed according to three GO categories, biological process (BP), molecular functions (MF), and cellular component (CC) (Fig. 8A, Supplementary Table S4). In module darkred, plenty of genes were related to transmembrane transport. For instance, amino acid transmembrane transport (GO:0003333) term was enriched in the BP category, and arginine transmembrane transporter activity (GO:0015181), L-lysine transmembrane transporter activity (GO:0015189), L-glutamate transmembrane transporter activity (GO:0005313), basic amino acid transmembrane transporter activity (GO:0015326) terms were enriched in MF category. In darkturquoise module, terms related to potassium ion transmembrane transport were enriched both in BP (GO:0071805) and MF (GO:0015079) categories, and some terms related to photosynthesis were enriched in BP (photosystem II assembly, GO:0010207) and CC (chloroplast envelope, GO:0009941) categories. In green module, still some photosynthesis associated GO terms were enriched, such as chlorophyll catabolic process (GO:0015996), chloroplast envelope (GO:0009941), chloroplast thylakoid membrane (GO:0009535).

To identify the hub genes in key modules, the coexpression network was analysed and visualized by Cytoscape, as shown in Fig. 8B. The genes in the network highly interconnected with others tend to be of great importance. The node size in the network represented the connection degree of genes, and the top seven genes with the highest connection that were considered hub genes were placed in the centre of the network.

Since the hub genes might be crucial in the drought stress response of peanut, we focused on these genes for further analysis. As shown in Table 1, several kinases were identified in the darkred module, including two LRR receptor-like serine/threonine-protein kinases (arahy.BD00MW, arahy.M9TY0X) and one brassinosteroid insensitive 1-associated receptor kinase (arahy.YY2SNP), which are LRR-RLKs. In addition, a serine/threonine-protein kinase and ribokinase isoform were identified. Genes in the darkred module were significantly upregulated in DT at 4 h and 8 h. In addition, genes encoding potassium transporter (KT, arahy.GBJ0AR and arahy.43ZW0S), pentatricopeptide repeat-containing protein (PPR, arahy.HSF0DX), E3 ubiquitin-protein ligase (arahy.D4PR4R), flavonoid 3&apos;monooxygenase (arahy.AM3FDB), and catalase were present in the darkturquoise module. In module green, genes were changed with a greater upregulation at 4 h and 8 h, especially in DT, such as genes encoding PPR (arahy.T7AVMG), aspartic proteinase (APA, arahy.Q8WU48), peroxisomal membrane protein (arahy.0QBZ7D), and cysteine synthase isoform X1 (arahy.AI24NX). In addition, an uncharacterized protein (arahy.CV4FMG) was identified.

**Table 1:**
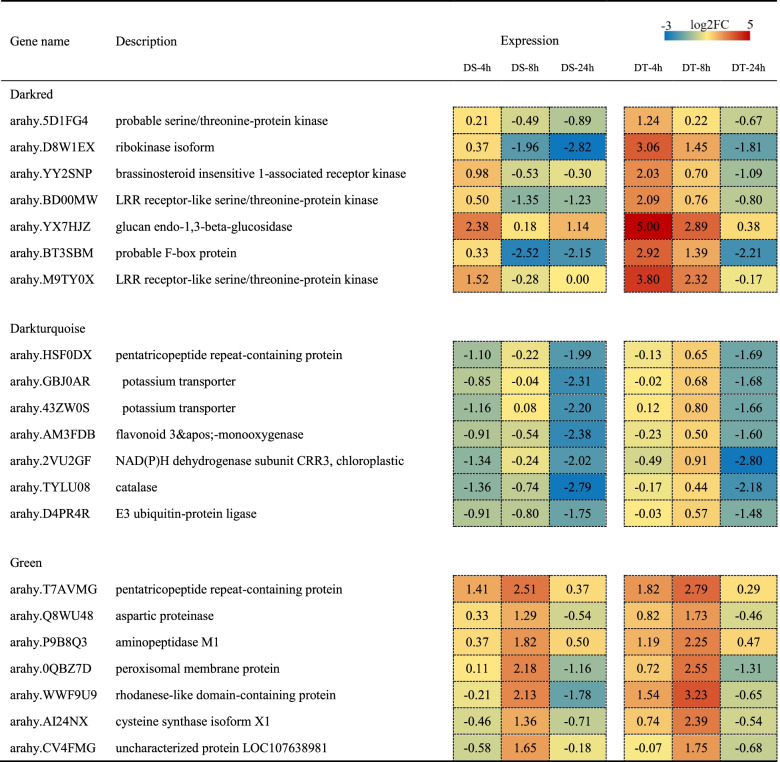
Differentially expressed hub genes identified in the key modules

## Discussion

Drought stress represents an important environmental limiting factor that affects agricultural production and induces a series of physiological and molecular responses in plants. In recent decades, considerable effort has been devoted to mitigating the possible adverse effects of drought. In this study, physiological responses and transcriptome data were examined and compared between drought-tolerant and drought-sensitive varieties, and integrated analysis of physiological traits and genes was performed using WGCNA.

First, accumulation of O_2_^•−^ was measured in peanuts. O_2_^•−^ is one of the ROS, which is considered to be incompletely reduced oxygen species [28]. O_2_^•−^, as a byproduct of abiotic stress, can act as a signaling molecule in various cellular responses [29]. However, current evidence suggests that excessive O_2_^•−^ accumulation has a deleterious effect on plant cells [30]. O_2_^•−^ is highly chemically reactive, and it can cause injury to intracellular proteins, lipids, nucleic acids, and organelles, ultimately leading to oxidative damage [31]. In this study, O_2_^•−^ accumulation could be observed in both drought-tolerant and drought-sensitive varieties after drought stress (Fig. 1A-B). However, vast discrepancies in TBARs content were observed between DS and DT. TBARs are widely believed to be products of polyunsaturated fatty acid peroxidation in response to oxidative stress, including malondialdehyde (MDA) and other minor aldehyde forms, which can reflect the degree of lipid peroxidation and membrane lipid injury [32, 33]. MDA represents a major constituent of TBARs. Abiotic stress, such as drought, cold and salt stress, induced an increase in MDA content in *Arabidopsis thaliana* [34]. The wild mungbean exhibited stronger drought resistance together with a lower level of MDA [35], indicating that drought-tolerant varieties exhibit a better ability to maintain stable TBARs content. In this study, DS had a significantly higher TBARs content than DT, especially at 24 h (Fig. 1C), indicating that DS sustained more damage during drought stress. This difference may be due to the differential response mechanisms, such as antioxidant capacity and osmotic adjustment ability.

Previous studies have demonstrated that the accumulation of osmotically active substances contributes to a decrease in osmotic potential, thereby maintaining cellular turgor, protecting the plasma membrane and improving cell water retention [36]. Proline is an osmotically active substance that connects proteins and water molecules via the hydrophobic and hydrophilic parts [37]. This phenomenon would prevent protein denaturation under drought stress, including antioxidative enzymes [38]. A higher T-AOC could be observed in DT, which may be associated with its higher proline content. In this regard, a study showed a higher proline level in the drought-tolerant peanut variety than in the drought-sensitive variety [39], consistent with the present study findings. Soluble sugars represent another osmotic adjustment substance that counteracts osmotic stress. An experiment showed that overexpression of the harpin-encoding gene in *Arabidopsis* mutants increased the contents of soluble sugar and proline, accompanied by improved drought tolerance [40]. The significantly higher contents of proline and soluble sugar in DT might confer stronger resistance to drought. In addition, it has been documented that osmotic adjustment substances contribute to sustaining photosynthesis under drought stress.

**Fig. 9:**
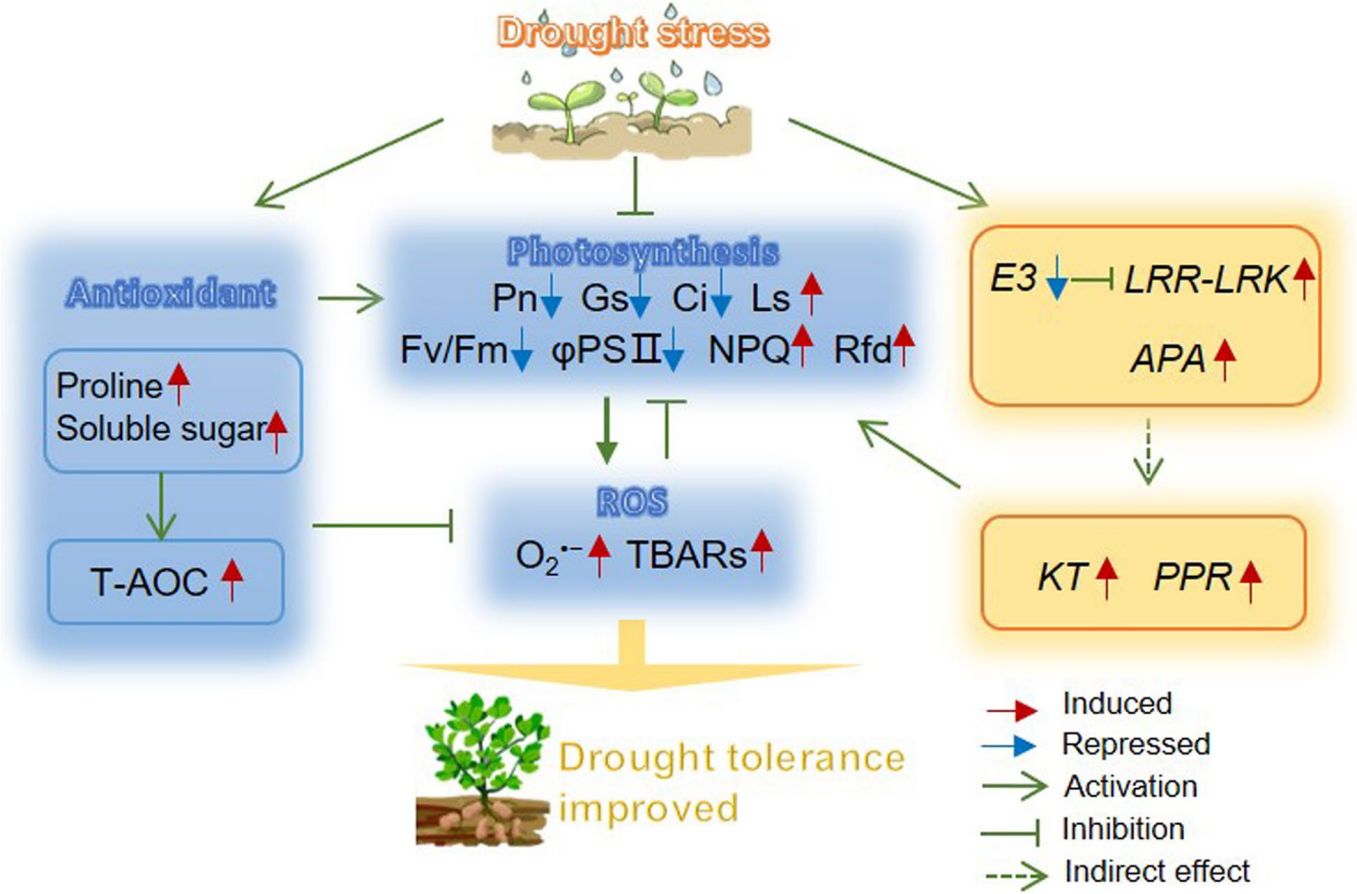
The possible regulatory network of peanut in response to drought stress. E3: E3 ubiquitin-protein ligase, LRR-RLKs: LRR receptor-like serine/threonine-protein kinase, APA: aspartic proteinase, KT: potassium transporter, PPR: pentatricopeptide repeat-containing protein

In the present study, we substantiated that drought stress significantly affected the photosynthesis rate in peanuts (Fig. 3). Inhibition of photosynthesis is one of the primary physiological pathways of drought stress, and the responses of photosynthesis to drought stress are extremely complex. In general, the effects of drought stress on plants are mainly due to stomatal limitation, followed by metabolic impairment [41]. As described in previous studies, photosynthesis rate reduction is mainly due to stomata for CO_2_/water exchange and photosynthetic activity in mesophyll cells [37, 42]. A study investigated the deleterious effect of drought stress on soybean photosynthesis and found that photosynthesis-related genes, including PS-II light harvesting complex-related genes, were significantly repressed [43]. Another study found that drought stress significantly affected the leaf chlorophyll content, net Pn, and stomatal conductance of wheat, and the Rubisco large and small subunits were downregulated in leaves [42]. Plants that sustain photosynthesis at lower water contents are manifestations of drought tolerance [44]. In the present study, there was a sustained increase in the Ls of peanuts under drought stress, together with a decrease in Gs, indicating that stomatal limitation is a major factor in photosynthesis reduction. It has been demonstrated that chlorophyll fluorescence is tightly associated with photosynthesis [45]. When exposed to drought conditions, excess light causes ROS production because it is less capable of thermal dissipation in PS II [46]. In turn, the accumulated ROS damaged the photosynthetic apparatus, especially PSII, and led to a decrease in PSII activity [47]. In barley, a high correlation was observed between Fv/Fm and photochemical events downstream of the PSII reaction centre in drought-treated plants [48]. Additionally, NPQ is conducive to dissipating radiation-free energy and avoiding the overexcitation of PSII [46]. Therefore, the higher NPQ values in the DT variety indicated a greater heat dissipation capability under drought stress, which was consistent with the lower O_2_^•−^ and TBARs contents (Fig. 9). The KEGG enrichment analysis revealed that a large number of DEGs were involved in the regulation of photosynthesis, indicating an association between genes and physio-biochemical traits.

To establish the association between genes and traits and to further analyse the regulatory mechanisms, a comprehensive WGCNA analysis was performed (Fig. 6). The darkred, darkturquoise, and green modules were considered key modules. Genes in these modules showed different expression patterns, which could explain the drought tolerance discrepancy between the two varieties. Genes in the key modules highly connected with others in the network were considered hub genes. It is widely acknowledged that hub genes form the backbone of the network and tend to be vital in specific physiological processes [49]. In the darkred module, there are four hub genes (arahy.5D1FG4, arahy.YY2SNP, arahy.BD00MW and arahy.M9TY0X) that are considered LRR-RLKs. LRR-RLKs play crucial roles in the plant stress response and are the largest subfamily of receptor-like kinases in plants [50]. They perceive signals, transduce the signal downstream, and finally phosphorylate specific substrates, thereby mediating cellular signalling transduction [51]. Early research demonstrated that LRR-RLKs are involved in dehydration early signalling [52]. In this study, LRR-RKs were significantly induced in DT at 4 h and 24 h, thus indicating their positive roles in the peanut drought stress response, especially at the early stage (Fig. 9).

In the dark turquoise modules, the hub genes included two *KT* genes. K^+^ participates in plant abiotic stress regulation by regulating stomatal movement, photosynthesis, and osmoregulation [53]. K^+^ transmembrane transport relies on potassium channels and transporters [54]. Moreover, research on barley revealed that overexpression of the *HvAKT1* gene improves drought tolerance, confirming the positive roles of the K^+^ transporter in drought [55]. The expression of *KT* genes (arahy.GBJ0AR and arahy.43ZW0S) in peanut were downregulated, especially in DS, indicating that drought stress disturbed the ionic regulatory capacity. In addition, we found that the E3 ubiquitin-protein ligase was downregulated in peanuts. The negative function of E3 ubiquitin-protein ligase has been established in various species. For instance, *PUB11*, *PUB22* and *PUB23* negatively regulate drought tolerance by degrading receptor-like protein kinases and ABA receptors in *Arabidopsis* [56, 57]. Moreover, *PUB27* in potatoes negatively regulates drought tolerance by mediating stomatal movement [58].

Additionally, two *PPR* genes (arahy.HSF0DX and arahy.T7AVMG) were identified in the darkturquoise and green modules. PPR is one of the largest protein families and contains helical repeat proteins that bind RNA and affect gene expression in mitochondria and chloroplasts [59]. The central roles in modulating the stability and translation of specific chloroplast mRNAs of *PPR10* have been confirmed in maize [60]. The *PPR* gene in the green module was markedly upregulated in DT (Table 1), heralding its potential contribution to chlorophyll biosynthesis and photosynthesis (Fig. 9). In addition, the *APA* in the green module was upregulated in the two varieties and was higher in DT. In *Arabidopsis*, overexpression of the *APA1* gene confers stronger drought tolerance [61], and overexpression of *ASPG1* improved drought avoidance through abscisic acid signalling [62]. Therefore, the upregulation of *APA* tended to positively regulate peanut drought tolerance in this study. Accordingly, a potential positive regulatory network of peanut drought tolerance was proposed, as shown in Fig. 9.

## Conclusion

Taken together, a comparative physiological analysis of peanut revealed the different response mechanisms between drought-sensitive and drought-tolerant varieties. Drought stress strongly inhibited photosynthesis and caused ROS accumulation in DS, leading to severe membrane lipid peroxidation. In contrast, the tolerant variety induced powerful antioxidant capacity by increasing the T-AOC, flavonoid contents, and osmotic adjustment substances under drought conditions. *LRR-RLKs*, *APA*, and *E3 ubiquitin-protein ligase* participated in peanut drought stress response by mediating cellular signalling transduction, and *KT* and *PPR* were upregulated, contributing to the maintenance of photosynthesis. The hypothesis of these processes is shown in Fig. 9 and will be explored in a future study.

## Materials and methods

### Plant materials and growth conditions

In this study, two peanut varieties, i.e., NH5 (bred by Shenyang Agricultural University) and FH18 (bred by Aeolian Sand Research Institute of Liaoning Academy of Agricultural Sciences), were selected as the drought-tolerant and drought-sensitive varieties, respectively, as described in a previous study [63]. The sterilized seeds were soaked in distilled water for 8 h and put in the dark at 25 °C for 24 h for pregermination. Germinated seeds were selected and sown in pots filled with clean river sand. Then, the seeds were grown in a growth chamber for 2 weeks and watered every 2 days with 1/2-strength Hoagland’s solution. The environmental conditions were 28/25 °C with a light/dark cycle of 16/8 h, relative humidity of 70%, and a light intensity of 500 μmol/m^2^/s. Two-week-old seedlings of two peanut varieties were gently uprooted and placed in sterile water for 24 h to adapt to hydroponic conditions. PEG-6000 (20%) was used to simulate drought conditions. The antepenult leaves were collected at 0 h (CK), 4 h, 8 h, and 24 h. The samples were frozen in liquid nitrogen and then stored at − 80 °C.

### Determination of drought stress-induced histochemical and physiological changes

Staining with NBT was used to detect O_2_^•−^ in peanut leaves. In brief, fresh leaves were vacuum-infiltrated in 0.1% NBT for 10 min and boiled with ethyl alcohol to remove the chlorophyll [64]. The samples were recorded by a digital camera. The O_2_^•−^ contents and T-AOC were measured by a Superoxide Anion Assay Kit (Solarbio, Beijing, China) and Total Antioxidant Capacity Assay Kit (Solarbio, Beijing, China). TBARs were extracted with 10% trichloroacetic acid and centrifuged at 12,000 rpm for 20 min at 4 °C. Then, the supernatant was mixed with 0.2% thiobarbituric acid and incubated at 95 °C in a water bath for 30 min. The mixture was subsequently cooled rapidly and centrifuged again. The absorbance was detected with a microplate reader at wavelengths of 532 and 600 nm [65]. The soluble sugar was detected according to the anthrone colorimetric method, and the absorbance of the mixture was recorded at 625 nm [66]. The flavonoid was extracted by ethanol, and the supernatant was mixed with 5% sodium selenite, 10% aluminium nitrate and 4% sodium hydroxide. The mixture was determined with colorimetry at 510 nm [67]. Then, 5 mL 3% sulfosalicylic acid was used to extract proline, and 2 mL supernatant was transferred to a clean tube and mixed with ninhydrin (47 mM), phosphoric acid (0.8 M), and glacial acetic acid (0.25 M) and subsequently boiled at 98 °C for 60 min. Finally, the mixture was extracted with 4 ml toluene, and the absorbance was recorded at 520 nm [68]. All of these experiments were repeated three times.

### Photosynthetic and chlorophyll fluorescence parameters

The Pn, Gs, and Ci were measured using CIRAS-2 (PP Systems, Hitchin, UK). The leaf chamber conditions were as follows: a PPFD of 1200 μmol m^− 2^ s^− 1^, relative humidity of 70%, a leaf temperature of 25 °C, and a CO_2_ concentration of 380 μmol mol^− 1^ in the leaf chamber.

After pretreatment in the dark for 30 min, the chlorophyll fluorescence parameters of leaves were detected and imaged using a Chl fluorescence imaging system (FluorCam FC800, Photon Systems Instruments, Brno, Czechia), including the Fv/Fm, the ΦPSII, the NPQ, and the Rfd. All of these experiments were repeated three times.

### RNA-seq library construction and transcriptomic data processing

Total RNA was extracted from samples using TRIzol reagent (Invitrogen) according to the manufacturer’s instructions. The high-quality RNA samples were used for cDNA library construction. Based on sequencing by synthesis technology, the cDNA library was sequenced by an Illumina high-throughput sequencing platform (Illumina, San Diego, California, USA). The sequencing data are available in the NCBI database under SRA accession number PRJNA657965. After removing the low-quality reads, the clean reads were obtained and mapped to the reference genome (https://www.peanutbase.org/data/public/Arachis_hypogaea/Tifrunner.gnm1.KYV3/arahy.Tifrunner.gnm1.KYV3.genome_main.fna.gz). FPKM was used to measure gene expression, and |log2 Fold Change (FC) | ≥ 1.00 and FDR ≤ 0.05 were used as standards for differentially expressed gene identification.

KEGG pathway analysis was performed based on the KEGG database. GO functional enrichment and classification analyses were carried out using the online tool agriGO.

### Quantitative real-time PCR analysis

Total RNA was extracted from peanut leaves using TRIzol. Reverse transcription was conducted using the Prime Script™ RT Master Mix Kit (TaKaRa, Dalian, China). qRT–PCR was performed using SYBR Premix Ex Taq™ kit (TaKaRa, Dalian, China) according to the manufacturer’s protocol. The 10-μL reaction contained 1.0 μL of cDNA, 0.2 μL of each primer, 3.6 μL of ddH_2_O and 5.0 μL of SYBR. The reactions were conducted as follows: 95 °C for 60 s, followed by 40 cycles of 95 °C for 15 s, 55 °C for 30 s and 68 °C for 30s. Actin11 (NCBI accession number: GO264911) was used as an internal control. The relative expression levels were calculated according to the Eq. 2^-ΔΔCT^. All the primers are listed in Supplementary Table S5.

### Co-expression network construct and analysis

An R package for WGCNA was used to construct the gene coexpression network [23]. Genes with FPKM≥1 were selected for subsequent analysis. The WGCNA network was constructed using a topological overlap matrix (TOM). The hierarchal clustering tree was defined by the dynamic hybrid tree cut algorithm. A power value of 7, minimum module size of 30 and minimum height for merging modules of 0.25 were used. The coexpression relationships in modules were analysed and visualized by Cytoscape v3.7.2 [69].

### Statistical analysis

All of the physiological data were statistically analysed with Microsoft Excel (Microsoft Corporation, USA) and SPSS 22 (SPSS Inc., USA), and graphs were constructed using GraphPad Prism 8 (GraphPad Software, Inc.). Student’s t test was conducted to compare between two varieties at the same treatment point, and * and ** represent significant differences at the 0.05 and 0.01 levels, respectively. The data between different time points were analysed by one-way ANOVA, and the least significant difference (LSD) test was used for multiple comparisons.

## Supplementary Information

**Additional file 1.**

**Additional file 2.**

**Additional file 3.**

**Additional file 4.**

**Additional file 5.**

**Additional file 6.**

## Data Availability

The sequence data is deposited in NCBI database under SRA accession: PRJNA657965 (https://www.ncbi.nlm.nih.gov/sra/PRJNA657965).
