# Supplementary material for: Comparative physiological and coexpression network analyses reveal the potential drought tolerance mechanism of peanut
Source: BMC Plant Biol. 2022 Sep 26;22:460. doi: 10.1186/s12870-022-03848-7 (PMC9511739; doi:10.1186/s12870-022-03848-7)
Supplement: Supplementary file 6 — Additional file 6. [file 12870_2022_3848_MOESM6_ESM.docx]

**Comparative physiological and co-expression network analyses to provide hub candidates of drought tolerance in peanut**


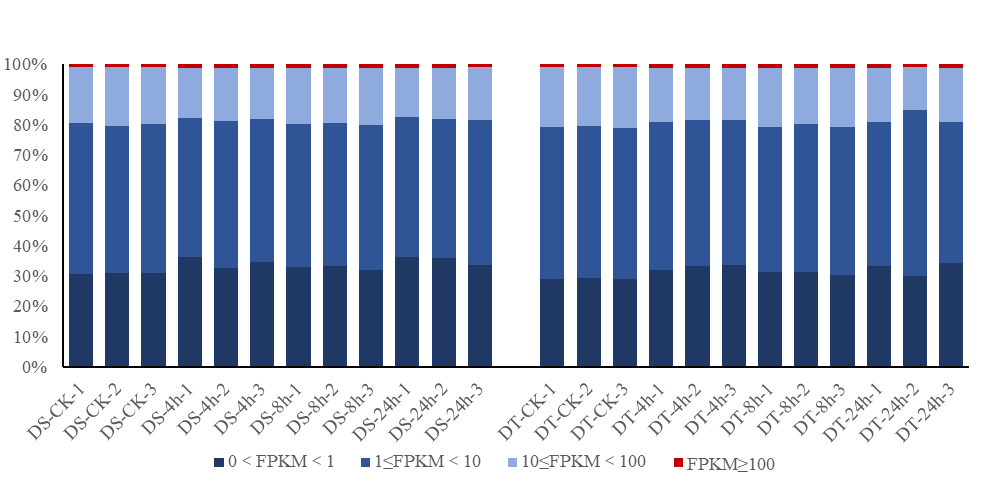
 Supplementary Figure S1 Numbers of expressed genes in each sample in two peanut varieties.


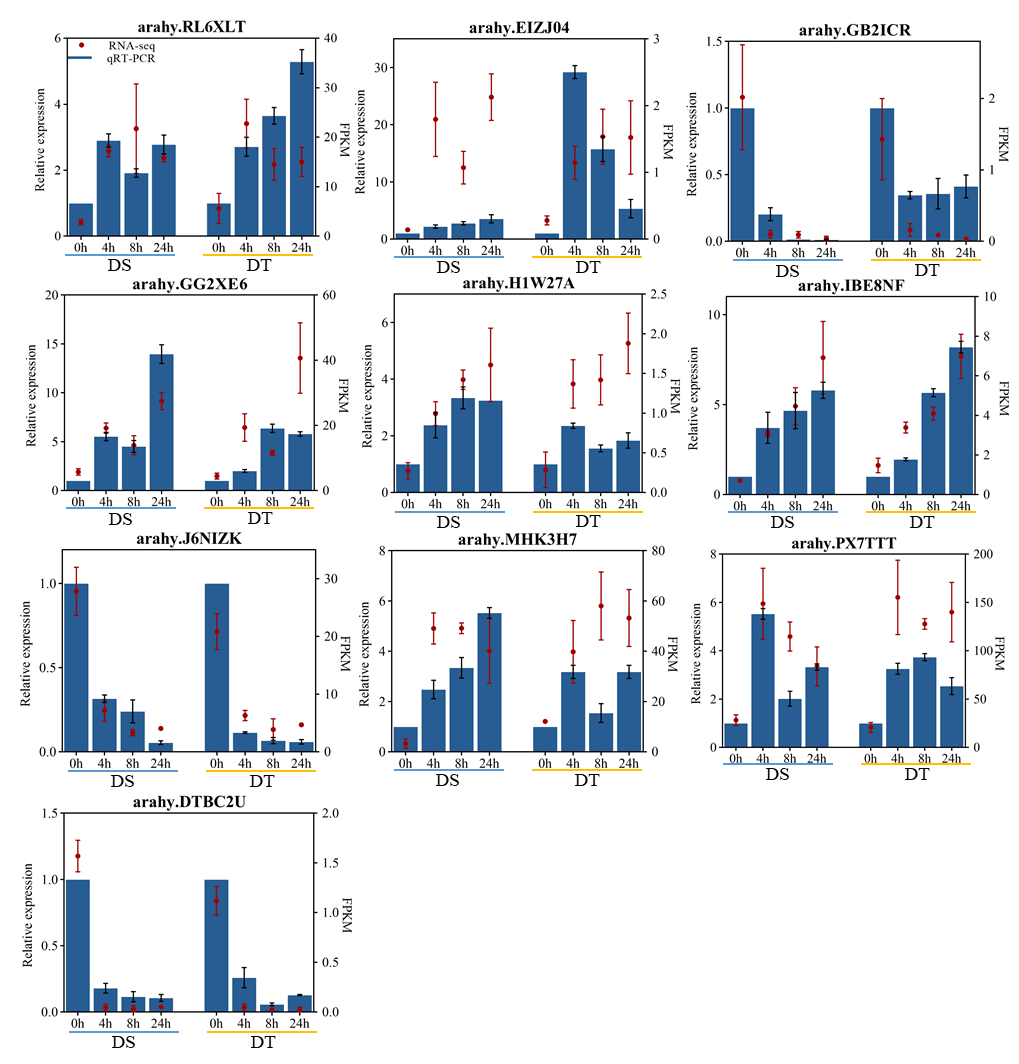


Supplementary Figure S2 Validation of differentially expressed genes in the RNA-Seq database by quantitative real-time PCR


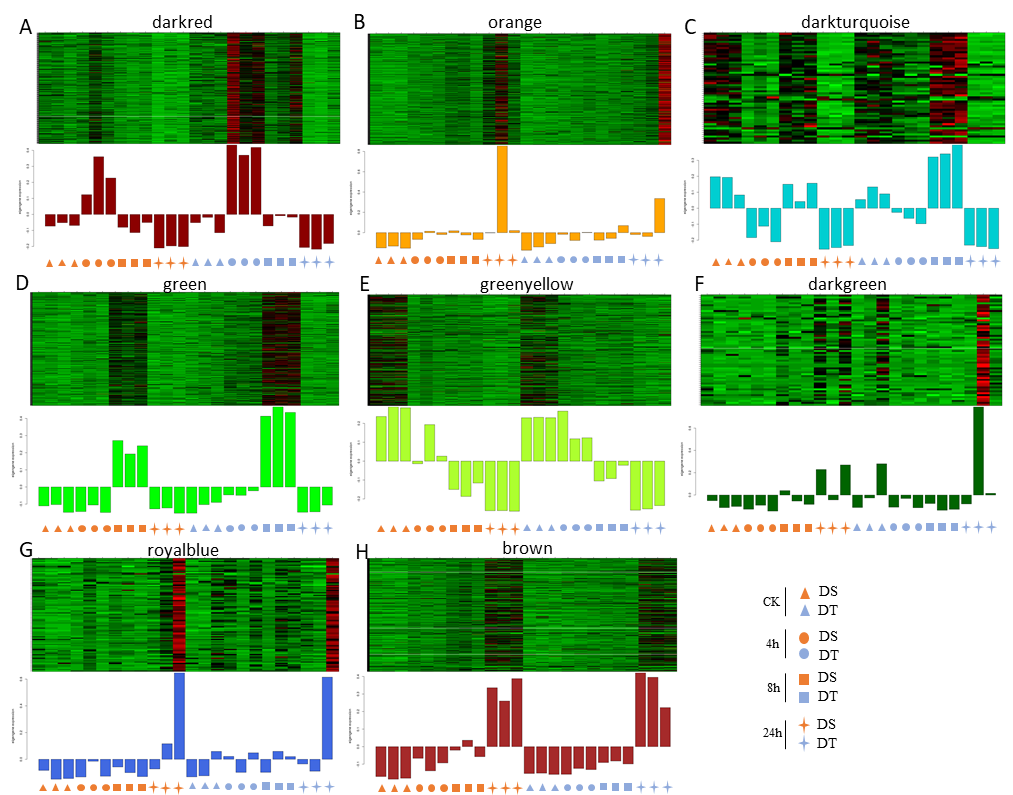


Supplementary Figure S3 Expression profiles of genes in nine co-expression modules with biological significance. (A) The darkred module; (B) The orange module; (C) The darkturquoise module; (D) The green module; (E) The greenyellow module; (F) The darkgreen module; (G)The royalblue module; (H) The brown module. Heatmaps show the expression profiles of all the co-expressed genes in each module. Bar graphs show the expression pattern of module eigengenes in each module.
